# 

**Published:** 1905-05-15

**Authors:** 


### CONTENTS.

Event and Comment - - - - - - - - - - 217

The People versus the Dentist, - - By Geo. Zederbaum D.D.S. 224

Cleaning Teeth, - - - - - - By N. S. Hoff, D.D.S. 233

Why use Low-carat Solder on New Dental Work, By William H. Trueman, D.D.S. 242

Gold Inlays, - - - - - - By E. H. Allen, D.D.S. 247

Announcements - - - - - - - - - - 250

Bibliography - - - - - - - - - - - 258

Obituary - - - - - - - - - - - 260

Indents - - - - - - - - - - - 261

